# Psoriasis Patients Treated With Methotrexate Have an Increased Risk of Nonmelanoma Skin Cancer: A Systematic Review and Meta-Analysis

**DOI:** 10.7759/cureus.37174

**Published:** 2023-04-05

**Authors:** Margaret E Lang Houser, Jacob R Stewart, Jerry D Brewer

**Affiliations:** 1 Dermatology, University of Michigan, Ann Arbor, USA; 2 Dermatology, Mayo Clinic, Rochester, USA

**Keywords:** squamous cell carcinoma, psoriasis, nonmelanoma skin cancer, methotrexate, incidence, basal cell carcinoma

## Abstract

Both psoriasis and methotrexate are associated with an increased risk of nonmelanoma skin cancer. The effect of methotrexate on the development of nonmelanoma skin cancer in patients with psoriasis is currently unknown. To evaluate this relationship, a systematic review of the literature was conducted using databases including Ovid Medline (from 1946), Scopus (from 1970), and Embase (from 1974) through June 2019. Observational comparative and case-control studies comparing psoriasis patients treated with methotrexate to those not treated with methotrexate with data on the subsequent development of nonmelanoma skin cancer in both cohorts were included based on prespecified criteria. Two reviewers analyzed all studies for relevant data, which were analyzed using OpenMeta-Analyst statistical software. Quality was assessed with the Newcastle-Ottawa method. Nine cohort and case-control comparative studies of 1,486 screened abstracts met the inclusion criteria. Of 11,875 reported patients with psoriasis, 2,192 were taking methotrexate. A meta-analysis demonstrated an odds ratio of 2.8 (95% confidence interval = 1.47-5.39; p = 0.002) for nonmelanoma skin cancer development in patients with psoriasis taking methotrexate compared with those not taking methotrexate. Based on these findings, psoriasis patients treated with methotrexate are at a significantly increased (2.8 times higher) risk of developing nonmelanoma skin cancer. Risk counseling can improve healthcare outcomes in patients with psoriasis.

## Introduction and background

Psoriasis is a chronic, systemic, dermatologic autoimmune disease associated with inappropriate thickening of the epidermis. This leads to the progressive formation of plaques and eventual disruption of the skin barrier [1-3]. The nails and joints are frequently involved [1-3]. Histologically, psoriasis presents with epidermal hyperkeratosis and acanthosis with epidermal inflammatory infiltrates [1]. Clinically, there are five main subtypes, namely, plaque, inverse, pustular, erythrodermic, and guttate [1,2]. All subtypes of psoriasis are associated with numerous comorbid conditions [1-5], including an increased risk of cardiovascular disease, metabolic syndrome, mental health diseases, and inflammatory bowel disease, among others [1-6]. Notably, psoriasis also carries an increased risk of solid organ cancers, lymphoproliferative diseases, and skin cancers [4,7-9]. The risk of skin cancer development is currently not well quantified [4,7-10].

The correlation between overall cancer risk and psoriasis increases with psoriasis severity throughout all age groups [7,9]. Some studies have suggested that this risk may be comparable to cancer risk in patients who have received organ transplants [7,9]. Among these cancers associated with psoriasis, nonmelanoma skin cancers (NMSCs) are frequent [8-17]. The risk is further increased for patients who are receiving systemic oral immune-regulating therapies [7,10,12,18].

For patients with psoriasis, the effects of many systemic therapies on NMSC risk are currently unquantified [10,12]. One primary treatment option for moderate-to-severe psoriasis is methotrexate (MTX) [3,9-11,19]. MTX is an antineoplastic and anti-inflammatory antifolate medication that acts by reversibly inhibiting dihydrofolate reductase, an enzyme required for adequate cell division [12,20]. It is used in many autoimmune conditions to control systemic inflammation [11,12,19,20]. Patients using MTX as a systemic treatment for moderate-to-severe psoriasis have been shown to have an increased risk of NMSCs. This risk has been demonstrated for MTX used as monotherapy and in combination with other treatments, as well as when used for other autoimmune disorders [9,11,12,14-16,19,20]. Moreover, this increased cancer risk may be greater with prolonged MTX usage [6].

Although the use of MTX has been associated with NMSC development, the extent of the NMSC risk associated with MTX therapy in patients with psoriasis is not well quantified [10,12,14-17]. Given the high frequency with which MTX is used in patients with psoriasis, this lack of conclusive data about the actual risk represents a gap in our knowledge and affects a large population of patients who could potentially benefit from more comprehensive counseling [14-17]. Therefore, this systematic review and meta-analysis aimed to clarify the risk of NMSC in patients with psoriasis treated with MTX. Given the chronicity of psoriasis, systemic therapy can be frequent and for long durations [10,12]. Thus, it is necessary to understand the effect of commonly used medications such as MTX on NMSC development. This improved understanding can influence education and skin cancer surveillance in these patients and ultimately decrease morbidity, possibly mortality, and healthcare costs.

## Review

Methods

This study was performed in accordance with prespecified criteria regarding study selection, exclusion criteria, search strategy, data extraction, and statistical analysis, as well as heterogeneity and measurement of inconsistencies. The methods used were in accordance with the Cochrane collaboration guidelines (www.cochrane.org), the Newcastle-Ottawa method of quality assessment, and the QUOROM statement [21-23].

Search Strategy

The search strategy for this systematic review, conducted by a medical librarian, included pooling of studies from multiple international databases, including Ovid MEDLINE (from 1946), Scopus (from 1970), Cochrane Central Register of Controlled Trials (from 1974), and Embase (from 1974) through June 2019. Search terms included “psoriasis,” “methotrexate,” “nonmelanoma skin cancer,” “skin tumor,” “skin cancer,” “skin carcinogenesis,” and “skin carcinoma,” as well as derivatives of these terms. All studies that met these search criteria were included for assessment. Reference lists were also evaluated for potentially relevant articles. Studies were further limited to those written in the English language and human studies. Published abstracts were included if all required data were present. To effectively broaden the search, we performed subgroup analyses that included multidrug/multitherapy regimens with MTX as a treatment. Patients with any history of skin cancer were also included.

Study Selection

Studies included in this review were prospective or retrospective observational comparative studies and case-control studies. No other types of articles including randomized controlled trials met the required criteria. Accepted two-arm studies included comparisons of psoriasis patients treated with MTX versus no therapeutic regimen or regimens other than MTX and reported the subsequent development of NMSCs. Psoriasis patients treated with MTX with any combination of therapies were included. Notably, for included case-control studies, if controls in the original data set were patients without psoriasis, then data were extracted from patients with psoriasis (who were considered cases) who were treated with medication regimens other than MTX. These data were then included in the comparison arm comprising patients with psoriasis treated with a medication other than MTX and assessing the subsequent risk of NMSC development. Studies were assessed for eligibility by two independent investigators (MEL and JRS), with any discrepancies resolved by unanimous decision, and a third investigator (JDB). Reviewer agreement was determined with the kappa (κ) statistic.

Study Quality Assessment and Risk of Bias

All studies used in the final analysis were assessed. We investigated the comparability of outcome assessments for each study. We followed the Newcastle-Ottawa quality assessment tool for observational cohort and case-control studies to evaluate the overall risk of bias [23-25].

Data Abstraction and Management

The primary outcome variable for this systematic review and meta-analysis was the development of NMSC in psoriasis patients treated with and without MTX. This assessment was reviewed by two independent investigators (MEL and JRS). Between the two reviewers, opinions differed regarding the inclusion of five studies for further evaluation. These articles were discussed until an agreement was made. The κ statistic for this study, calculated using GraphPad software 25, was 0.918 (standard deviation (SD) = 0.021; 95% confidence interval (CI) = 0.89-0.96), which indicated a high level of reviewer agreement.

Data were abstracted from the final studies agreed upon by the two independent reviewers. Data abstracted from these studies included the number of patients treated with MTX, the number of patients not treated with MTX, and the number of patients who developed NMSC in each category. No data were missing from the accepted studies. All pertinent data were analyzed using OpenMeta-Analyst statistical software [26]. P-values <0.05 were considered significant.

Subgroup Analysis and Assessment of Heterogeneity

The I^2^ statistic was used to quantify the heterogeneity of pooled studies. Heterogeneity was defined as the variation in the effect estimates. I^2^ values of 0% to 25% were considered low heterogeneity, 25% to 50% moderate heterogeneity, and greater than 50% high heterogeneity [24]. When found, high heterogeneity was believed to result from high variation within the pooled studies that were included.

Results

Studies Included

**Figure 1:**
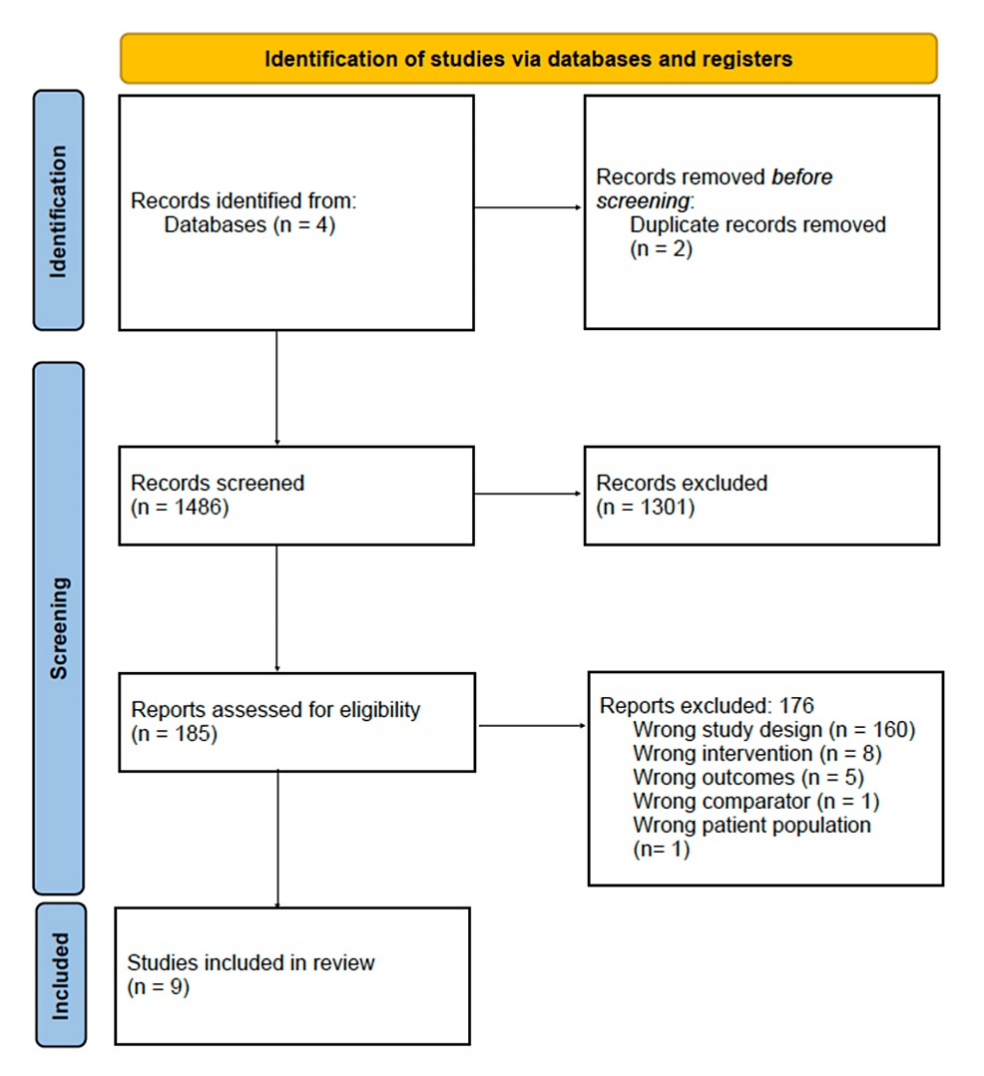
PRISMA flow diagram for study selection. PRISMA = Preferred Reporting Items for Systematic Reviews and Meta-Analyses

A total of 1,485 studies were considered for inclusion based on the initial review of the literature. One additional article was found through an external review of the literature, for a total of 1,486 studies (Figure 1).

After a review of abstracts, 185 studies were included for full-text review. Of these articles, 176 were excluded. This resulted in nine articles that met the inclusion criteria (Table 1) [27-35]. Of the 185 studies included for a full review, there were no disagreements regarding final study inclusion between the independent evaluators.

**Table 1:** Summary of included studies [27-35]. NMSC = nonmelanoma skin cancer; MTX = methotrexate; CI = confidence interval

		Number with NMSC development/Group total		
Study	Year	MTX users	Non-MTX users	Odds ratio (95% CI)
Cohort studies				
Staumont-Sallé et al. [27]	2012	0/267	1/131	0.17 (0.01-4.03)
Costa et al. [28]	2016	2/192	4/426	1.11 (0.24-6.00)
Lange et al. [29]	2016	58/375	1/30	4.15 (0.60-28.98)
Saliba et al. [30]	2016	1/106	61/6,350	0.64 (0.03-15.19)
DeShazo et al. [31]	2017	11/432	61/6,350	2.61 (1.38-4.92)
Case-control studies				
Stern et al. [32]	1982	80/606	0/297	69.84 (4.35-1,122.48)
Mali-Gerrits et al. [33]	1991	11/134	4/93	1.84 (0.60-5.61)
Bhate et al. [34]	1993	4/50	30/2,197	5.50 (2.01-15.06)
Hannuksela-Svahn et al. [35]	2000	5/30	11/137	1.92 (0.71-5.18)

Patients

The nine articles that met the inclusion criteria for this meta-analysis included a total of 11,875 unique patients. Of these patients with psoriasis, 2,192 were taking MTX and 9,683 were not taking MTX. NMSC developed in 172 patients taking MTX (7.8%) and 112 patients not taking MTX (1.2%). Of note, patients not taking MTX were potentially using various other treatment regimens.

Subgroup Analysis and Assessment of Heterogeneity

By forest plot analysis, the overall heterogeneity of the included studies was 42.6%, consistent with moderate heterogeneity, and was not statistically significant (p = 0.08). Therefore, all studies were included for final data analysis.

Data Synthesis

**Figure 2:**
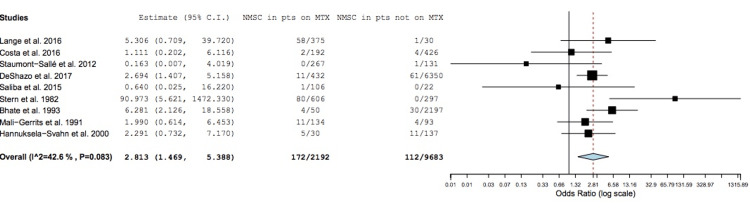
Forest plot analysis of included studies. Odds of developing NMSC in patients with psoriasis treated with MTX compared to patients with psoriasis not treated with MTX; black horizontal lines, 95% CIs for individual studies; black vertical line, lack of statistical significance; red vertical line, the overall OR for all studies; blue diamond, the overall effect for all studies. CI = confidence interval; NMSC = nonmelanoma skin cancer; pts = patients; MTX = methotrexate; OR = odds ratio

Patients with psoriasis taking MTX had a higher risk of NMSC development than those not taking MTX (estimated odds ratio (OR) = 2.8; 95% CI = 1.47-5.39; p = 0.002); heterogeneity was moderate, as stated (I^2^ = 42.6%; p = 0.08) (Figure 2) [27-35].

Risk of Bias

Independent quality assessment yielded two discrepancies between reviewers. These differences did not affect either study’s overall risk of bias. Thus, ultimately, there was agreement in the overall categorical rating of each article. As agreed upon by both evaluators, all studies were deemed high quality and demonstrated a low risk of bias based on the Newcastle-Ottawa Scale (Tables 2, 3) [27-35].

**Table 2:** Study quality characteristics of case-control studies [32-35] (According to Newcastle-Ottawa guidelines [23]). ^a^: Total score refers to the risk of bias: 7-9, low risk; 5-6, moderate risk; <5, high risk.

Study	Case definition adequate	Representative of cases	Selection of controls	Definition of controls	Comparability on the basis of design	Ascertainment of exposure	Same method of ascertainment	Nonresponse rate	Total^a^
Bhate et al. [34]	1	1	0	1	2	1	1	0	7
Stern et al. [32]	1	1	0	1	2	0	1	1	7
Hannuksela-Svahn et al. [35]	1	1	1	1	2	1	1	1	9
Mali-Gerrits et al. [33]	1	1	1	1	2	1	1	0	8

**Table 3:** Study quality characteristics of cohort studies [27-31] (According to Newcastle-Ottawa guidelines [23]). ^a^: Total score refers to the risk of bias: 7-9, low risk; 5-6, moderate risk; <5, high risk.

Study	Representativeness of the exposed cohort	Selection of nonexposed cohort	Ascertainment of exposure	Demonstration that outcome of interest was not present at the start of the study	Comparability based on design	Assessment of outcome	Follow-up long enough for outcomes to occur	Adequacy of follow-up of cohorts	Total^a^
Lange et al [29]	1	1	1	0	2	1	1	1	8
Costa et al [28]	1	1	1	1	2	1	1	0	7
Staumont-Sallé et al [27]	1	1	1	1	2	1	1	1	9
DeShazo et al [31]	1	1	1	1	2	1	1	1	9
Saliba et al [30]	1	1	1	1	2	1	1	1	9

Additional Analysis

We subsequently analyzed and compared the results for cohort and case-control studies independently. For cohort studies alone, patients with psoriasis taking MTX had a higher risk of NMSC development than those not taking MTX, although it was not statistically significant (OR = 2.00; 95% CI = 0.91-4.35; p = 0.08); heterogeneity was low (I^2^ = 16.82%; p = 0.31). In contrast, case-control studies alone showed a significantly higher risk of NMSC development for patients with psoriasis taking MTX versus not taking MTX (OR = 4.54; 95% CI = 1.52-13.54; p = 0.007), although heterogeneity was high (I^2^ = 61.25%; p = 0.05).

Discussion

Psoriasis patients treated with MTX have a 2.8 times higher chance of developing NMSC compared to those who were not treated with MTX. Patients with psoriasis who will potentially be on systemic treatment for many years could have a substantially increased risk of skin cancer. Several factors help determine the correct systemic medication in patients with psoriasis; however, this information should be taken into consideration. Increased awareness of an increased risk of NMSC in patients with psoriasis treated with MTX can decrease morbidity and mortality.

The findings of this meta-analysis highlight the need to provide thorough patient education on the risks of skin cancer development, especially in patients treated with MTX. Although skin cancer prevention is an important discussion for all patients, educating those with psoriasis about the increased risk of skin cancer in the setting of MTX can potentially affect tumor burden and patient comorbidity from NMSC. Risk modification can include increased screening for skin cancer with regular full-body skin examinations, more thorough counseling on appropriate skin protection, and increased education about signs of NMSC.

To our knowledge, this systematic review and meta-analysis encompass the most comprehensive data to date on the risk of NMSC in patients with psoriasis treated with MTX. However, the limited number of high-quality investigational studies analyzing this association between MTX, psoriasis, and NMSC indicates that further research is needed. Nevertheless, the high quality of data in the included studies and the low risk of bias given the study designs are strengths of this review.

Of note, multiple studies in the initial literature review did not specify the skin cancers included in the study as melanoma or NMSC. For this reason, these studies were excluded because our primary interest was identifying the risk of NMSC development. Further evaluation of these studies can potentially yield additional information about this subject.

Another limitation of this review was the inclusion of all patients taking MTX, regardless of other regimens they were using concurrently, including other nonbiologic systemic medications, phototherapy, and biologics. We believed that including these patients was necessary to have a complete understanding of the effect of MTX on NMSC risk for patients with psoriasis and would add to the power to address the question. For this reason, however, we cannot say that the findings reported in this study represent the sole effect of MTX on NMSC development because we could not estimate the role of other therapies in the development of skin carcinomas in patients using multidrug regimens. In fact, the risk of NMSC may be higher than identified in this study in psoriasis patients treated with MTX compared with no systemic therapy, given that many systemic therapies in the comparative arm have also been associated with NMSC development.

Notably, a recent meta-analysis showed that psoriasis patients treated with biologics did not have an increased risk of cancer overall [17]. Although previous studies have not quantified the risk associated with NMSC development in patients using MTX alone or in conjunction with other regimens, many have identified a potential relationship between systemic therapies used in psoriasis and an increase in NMSC risk [12-16,18]. Thus, our findings differ from those of multiple previous studies acknowledging that many other systemic regimens used for psoriasis may independently be associated with an increased NMSC risk [13-16]. The current meta-analysis indicates that biologics may have less of an effect on the risk of skin cancer development than originally thought [13-17].

Although the effects of combined psoriasis therapies are largely unstudied, the findings of this meta-analysis indicate a need for thoughtful patient counseling when prescribing MTX alone or in conjunction with other therapies. Because MTX has been used in the treatment of psoriasis for more than 50 years and is still frequently used, the information provided in this systematic review and meta-analysis is extremely useful when counseling patients beginning MTX regimens [2]. Future studies that further elucidate the effect of MTX on NMSC development could be beneficial.

## Conclusions

Patients with psoriasis who are treated with MTX have a 2.8 times higher risk of NMSC. These findings suggest a need for annual skin examinations, thorough counseling about sun protection, and education on the appearance and symptoms associated with NMSCs in this patient population. Counseling regarding these findings can decrease patient comorbidity from NMSC, improve healthcare outcomes, and decrease costs for patients with psoriasis.
